# Science diplomacy in the Global South: a case study of Brazilian participation in the PLATO mission

**DOI:** 10.3389/frma.2026.1856162

**Published:** 2026-08-06

**Authors:** Daniel Dalla Vecchia Gueter, Flavia Loss de Araujo, Alexandre Ramos Coelho, Rodrigo de Marca França, Vanderlei Cunha Parro

**Affiliations:** 1 Laboratory of Embedded Electronic Systems, Mauá Institute of Technology, São Caetano do Sul, Brazil; 2 FESPSP – São Paulo School of Sociology and Politics, São Paulo, Brazil; 3 Institute of Advanced Studies, University of São Paulo, São Paulo, Brazil; 4 FAAP – Armando Alvares Penteado Foundation, São Paulo, Brazil

**Keywords:** aerospace technology, Global South, PLATO mission, science and innovation, science diplomacy

## Abstract

Studies on Science and Innovation Diplomacy have become increasingly established as a subfield of International Relations, with growing attention devoted to the role of Global South countries in international scientific cooperation. This article examines the participation of a Brazilian university in the European Space Agency's PLATO mission to explore how large-scale scientific collaborations can foster science diplomacy while simultaneously revealing the institutional and structural constraints faced by emerging countries. Drawing on a qualitative approach and a literature review, the single-case study contributes to the Research Topic “Linking Knowledge Across Borders: Science Diasporas and Global South Development” analyzing how a Brazilian laboratory contributed to the mission between 2009 and 2024. Initially conceived as a scientific cooperation project, the initiative gradually came to involve an increasing number of Brazilian state actors, expanding its significance beyond its original technical objectives. The findings show mechanisms by which international collaboration strengthened institutional relationships and diplomatic engagement, constituting a case of Science for Diplomacy and enhancing Brazil's international visibility. At the same time, the case highlights how Global North–South asymmetries, governance arrangements, and domestic institutional limitations shape the opportunities and challenges of the Global-South participation in large-scale scientific and technological initiatives.

## 1 Introduction

When comparing the Global South and the Global North, asymmetries are present in most of the scientific spectrum. The socioeconomic disparities, mostly due to historic events such as colonization, lead to a sensible gap in technology innovation and academia breakthroughs. The path for South countries to achieve the same outcomes as North countries is usually way harder, but even with all the difficulties, there are several relevant scientific initiatives being conducted by the Global South. This established status quo is even more explicit in new research areas, such as Science Diplomacy, and in “Big Science” projects that require long-term and costly efforts to happen. Both examples were chosen purposely, since they are the main scope of this work.

Science Diplomacy, also widely referred as Science and Innovation Diplomacy (S&ID), is a relatively new research topic that studies the synergy between scientific goals and diplomatic relations, investigating how one impacts the other, and vice versa. “Big Science” are large-scale scientific projects that require a high number of interdisciplinary scientists and stakeholders, relying on a well structured organization and complex technology.

Facing the context given above, this paper aims to contribute to these subjects by analyzing the participation case of a Brazilian higher education institution in the PLATO (Planetary Transits and Oscillations of stars) space mission of the European Space Agency (ESA). Active between 2009 and 2024 and initially treated merely as scientific cooperation, the project involved an increasing number of Brazilian actors over time and also represented a closer relationship among the countries involved. Based on this perspective, this study seeks to answer the following question: how did this specific act of international cooperation become an example of science for diplomacy, bolstering Brazil's image in the international space sector? The single-case under analysis concerns the work of researchers and engineers from the Mauá Institute of Technology (IMT) in the ESA's PLATO satellite mission and was selected based on two criteria. The first one is that few Brazilian and Latin American higher education institutions participate in projects of this scale in the field of aerospace engineering. And the second is that it is even rarer for private Brazilian institutions to allocate resources to this type of research and to be integrated into international cooperation networks.

This national case study, and the discussion that follows it, are aligned with the Research Topic “Linking Knowledge Across Borders: Science Diasporas and Global South Development,” mainly addressing the topics of: knowledge circulation; technology transfer; science diplomacy; knowledge and power asymmetries in Global North-South collaboration; and the internationalization and visibility of science from the Global South. It also illustrates the perspective advocated by the S4D4C (2019) declaration, according to which scientific diplomacy goes beyond mere international scientific cooperation and may serve, directly or indirectly, as an instrument for advancing diplomatic objectives.

The research is exploratory and employs document analysis as its primary method, based on the hypothesis that the participation of a Brazilian higher education institution in the PLATO project contributed to broader objectives beyond scientific cooperation, strengthening Brazil's image in the international space domain. Considering that it's a single-case study, it's important to clarify that the hypothesis and its findings illustrate a possible mechanism through which scientific cooperation may evolve into diplomatic outcomes, rather than a generalized pattern demonstrated across different cases.

To address the subject, the article is structured into three sections, in addition to this introduction and the conclusion. The first section presents the conceptual debate on Science Diplomacy with the typology adopted, and introduces the case study of the IMT and its participation in the ESA's PLATO mission, demonstrating, through a timeline and document analysis, how international cooperation enabled technological, scientific, and diplomatic exchanges. The second section presents the results of the case in dialogue, highlighting positive implications for strengthening S&ID and fostering development in the Global South. The third and final section, prior to the conclusion, discusses the need for greater institutional support, structural asymmetries in North-South international scientific cooperations, and the importance of international cooperation among researchers in the S&ID field.

At its core, this article examines an example of S&ID related to cooperation between the Brazilian university IMT and the ESA. The case was selected due to the success of IMT's participation in a scientific cooperation project with ESA, a situation that presents several unique characteristics and challenges explored in this study, and that deviates from the typical pattern observed in Brazil and other Global South countries.

## 2 Methods

This study adopts a qualitative and exploratory approach, structured around a single case study of IMT's participation in ESA's PLATO space mission. The objective of the research is to understand how a long-term international scientific cooperation initiative can generate effects that extend beyond the technical and scientific sphere, contributing to institutional and diplomatic rapprochement between actors from the Global South and European space research institutions.

The case was selected intentionally due to its exceptional nature. The participation of a private Brazilian institution in a large-scale international scientific space mission represents a rare occurrence in the Latin American and Global South contexts, particularly in a sector characterized by high technological costs, strong dependence on public funding, and a concentration of scientific capabilities in developed countries. Furthermore, the case enables the observation of an international cooperation process developed over more than a decade, involving different types of actors, including researchers, universities, space agencies, governmental institutions, and funding agencies. This provides an opportunity to analyze scientific, institutional, and diplomatic interactions at multiple levels.

The research is primarily based on document analysis. The documents examined include one institutional letter, three memoranda of understanding, seven institutional recognition documents, and one funding record, materials related to the international cooperation developed between 2009 and 2024 among the partners of the PLATO mission. These documents were produced by Brazilian and European institutions directly or indirectly involved in the project, including IMT, ESA, the Brazilian Space Agency (AEB), the Ministry of Science, Technology and Innovation (MCTI), the French Space Agency (CNES), the German Aerospace Center (DLR), the Paris Observatory (OBSPM), and other institutions participating in the PLATO mission consortium.

The documents were selected according to three main criteria: (1) relevance to the trajectory of IMT's international cooperation within the PLATO mission; (2) evidence of institutional, scientific, or diplomatic interaction among the actors involved; and (3) direct connection to technical, institutional, or political milestones associated with the project's development. The document analysis sought to identify recurring patterns of interaction, mechanisms of international cooperation, processes of institutional trust-building, and evidence of rapprochement between scientific institutions and governmental actors.

To organize the data analytically, the events identified throughout the cooperation were classified into four categories: project milestones, related to the technical development of SimuCam and the evolution of the mission; institutional milestones, referring to funding, support, and institutional recognition; diplomatic milestones, associated with the rapprochement between Brazilian and European governmental actors; and new opportunity milestones, linked to the emergence of new international cooperation initiatives resulting from IMT's participation in the PLATO mission.

In addition to the document analysis, a structured timeline of the cooperation was developed and used as an analytical instrument to identify the evolution of interactions among the actors involved over time. The timeline made it possible to observe how relationships initially centered on technical and scientific demands gradually came to involve governmental institutions, space agencies, and broader cooperation initiatives. In this sense, the chronology was employed not merely as a historical description, but as a mechanism for identifying patterns of institutional expansion and potential diplomatic developments associated with scientific cooperation.

The data analysis was conducted through a qualitative and interpretative approach. The study sought to identify the mechanisms through which recurring technical interactions contributed to the construction of trust among partners, the expansion of international cooperation networks, and the rapprochement between Brazilian and European institutions. Accordingly, the research does not assume that all scientific cooperation automatically produces diplomatic outcomes; rather, it investigates how specific institutional and scientific dynamics may facilitate processes of international rapprochement and the creation of new spaces for dialogue between state and non-state actors.

The research nevertheless acknowledges important methodological limitations. As a single-case study, its findings cannot be generalized to all contexts of international scientific cooperation or science diplomacy in the Global South. Furthermore, a significant portion of the evidence analyzed consists of institutional documents produced by the actors involved in the project themselves, which may reflect pre-dominantly positive perspectives on the cooperation. Another limitation concerns the absence of formal interviews with mission participants, restricting access to individual perceptions and alternative interpretations of the process under analysis. Nevertheless, it is argued that the combination of document analysis, chronological reconstruction, and qualitative interpretation makes it possible to identify relevant elements for understanding the institutional and diplomatic impacts of international scientific cooperation involving Global South actors. This study seeks to contribute to a growing research agenda in this field and to encourage further investigations on the subject, including studies based on semi-structured interviews.

Since S&ID constitutes a very important concept for this work, the first part of this section reviews the evolution of the field, tracing its development from the earliest debates to more recent contributions. It also defines the typology and analytical framework adopted throughout the article, providing the conceptual basis for the discussion and interpretation of the single-case study presented in this work.

### 2.1 The science and innovation diplomacy framework guiding this study

Science Diplomacy has consolidated itself as a subfield of research within International Relations (IR) since the early 21st century, and although the field is strongly influenced by the Anglo-Saxon tradition and its main models are derived from the experiences of the Global North, particularly those of the United States (EUSDA, 2025), the literature on the subject has grown significantly in recent years in Latin America. Despite the inherent challenges faced by countries in the Global South in conducting scientific research and exploring new technologies, Latin American researchers have become relevant voices in this debate and have emphasized the importance of science diplomacy for the defense of national interests. Against this background, this subsection reviews the evolution of the Science Diplomacy literature and presents the conceptual framework that guides the analysis of this single-case study.

Scientifically relevant research, as a consolidated practice, is grounded in participation and peer review, mechanisms that ensure the quality of the knowledge produced. This process frequently transcends national borders, fostering international collaborations that involve both state and non-state actors, thereby strengthening relations among nations. Although technology transfer has been observed since earlier periods, it was in the 20th century that its importance received one of its first official recognitions. In 1945, the report Science, the Endless Frontier, submitted by the Director of the Office of Scientific Research and Development to the President of the United States, highlighted the strategic value of international exchange of scientific knowledge for national development (Bush, 1945).

From the Cold War period (1945–1989) onward, Western countries began to invest heavily in research and to compete for superior results. Extensive networks of researchers became common in addressing scientific challenges, supported by both public and private funding. From the perspective of states, investment in science does not merely translate into improved living conditions for humanity and economic strengthening, but also constitutes a power competition that involves their own security. More recently, technological competition has emerged as a central element in discussions on IR. Scientific cooperation, in its original form, is neutral and aimed solely at the advancement of science; however, it may also be employed to serve economic and political interests that shape relations among states, which use it as a diplomatic tool to garner support within the international system and to project a positive image. Today, science is one of the main instruments of soft power (Nye, 2004) available to states.

According to Turekian et al. (2015), international scientific cooperation and scientific diplomacy are overlapping activities: they are related, yet analytically distinct. International scientific cooperation is primarily concerned with advancing scientific discovery itself, whereas the central purpose of science diplomacy is often to use science to promote a state's foreign policy objectives or broader interstate interests. In other words, international scientific cooperation tends to be driven by individuals and groups, whereas science diplomacy, although it may stem from individual efforts, frequently involves state-led initiatives in the realm of scientific collaboration. Therefore, international scientific cooperation may or may not encompass science diplomacy. In the case of this study, science diplomacy is not understood as any sort of international scientific cooperation, but specifically as one of the outcomes of the continuous partnership among Brazilian and international institutes.

The topic began to be systematically studied in the 1990s, and first literature emerged in the early 21st century examining the intersection between science and foreign policy. In 2010, the Royal Society and the American Association for the Advancement of Science (AAAS) introduced the concept of scientific diplomacy The Royal Society and American Association for the Advancement of Science, 2010. Today, this field is organized around three main approaches, presented in Table 1: science in diplomacy, diplomacy for science, and science for diplomacy.

**Table 1:** The three approaches to science and innovation diplomacy.

Approach	Definition
Science in diplomacy	The use of scientific knowledge to inform the formulation and decision-making processes in foreign policy.
Diplomacy for science	The use of diplomacy between countries to enhance scientific outcomes through international collaboration.
Science for diplomacy	The application of scientific results derived from international cooperation as a tool to strengthen diplomatic relations among states.

This typology serves as a reference framework for identifying activities that connect diplomatic practices and science, and has, in fact, been challenged by several authors such as (Flink 2020) and Ruffini (2022). While it provides general guidance for the debate, the typology is both reductive and imprecise, potentially facilitating the instrumentalization of science to serve the specific interests of countries or individuals involved in funding research.

Moreover, the AAAS typology and much of the literature on science diplomacy overlook the asymmetries between the Global North and the Global South, defined in Mahler (2017), in the fields of science and innovation (Richards, 2025). These disparities, rooted in historical socioeconomic differences, become even more pronounced in the context of S&ID (Oppenheimer, 2016), given its relatively recent consolidation. The persistent under representation of Global South states in scientific discussions, coupled with the reproduction of coloniality in North–South relations, also permeates the field of S&ID (Polejack et al., 2022). However, these dynamics are not always adequately acknowledged in a literature that remains largely shaped by perspectives originating in the Global North. This imbalance is also reflected in the patterns of scholarly production. Rüland et al. (2023) identify a marked geographical imbalance in the production of S&ID scholarship. Although authors affiliated with African and Asian institutions have only become more prominent since 2023, the field has remained largely dominated by researchers based in European institutions, which have accounted for almost all published articles since 2014.

In the Latin American context, the focus of this article, obstacles such as political instability and ideological fragmentation limit the advancement of science diplomacy (CILAC and UNESCO, 2020). Although studies such as da Silva et al. (2021) highlight the gradual institutionalization of S&ID in the region and point to promising initiatives, there remains a need for greater integration between non-state actors, such as scientists, companies, and civil society organizations, and governmental structures responsible for enabling the internationalization of research. Echeverría King et al. (2021) presents a phenomenological analysis of science diplomacy based on the Colombian experience, examining the main country's science diplomacy practices and in the end proposing a governance framework tailored to emerging economies. Based on the empirical Colombian case analysis, the authors argue in the proposed governance framework that advancing the area of science diplomacy requires institutional mechanisms capable of bridging two traditionally distinct policy domains: science, technology, and innovation policy, and foreign policy.

Regarding the primary objectives of S&ID, important differences can be observed between the priorities of the Global South and those of the Global North. While Global North countries often utilize science diplomacy to facilitate international cooperation, mitigate diplomatic frictions, and enhance their soft power, Global South countries more frequently view S&ID as a mechanism for promoting national development Karacan and Ruffini, (2023). From this perspective, S&ID is often mobilized to strengthen scientific and technological capabilities, foster economic growth, and support broader efforts to reduce developmental gaps and achieve technological catch-up.

An interesting analytical model for understanding states' engagement with science diplomacy was proposed by Ruffini and Krasnyak (2020), who discuss the concept of S&ID from a realist-constructivist perspective focused on the nation-state and designed to be applicable to countries in the Global South. In this model, the authors identify three main motivations (called strategic drivers) for a state to invest in S&ID: international cooperation (the sharing of resources), access to knowledge, infrastructure, and funding, and influence, that is, the attempt to participate in decision-making processes on a wide range of issues in the international arena.

The model also distinguishes between Science and Technology (S&T) objectives, which are related to knowledge production and the strengthening of scientific capacities, and non-S&T objectives, which encompass political-diplomatic, economic, and development goals. Furthermore, the model systematizes the main instruments used by countries to operationalize their science diplomacy strategies, classifying them as strategic tools, which consist of policy documents produced by government agencies to achieve their policy objectives; operational tools, which refer to the human and financial resources provided by governments; and support tools, which include training activities and the dissemination of knowledge about S&ID, aimed at developing professionals capable of working in this field within a given country.

For the purposes of this study, the concept of science for diplomacy will be employed to analyze the case study presented. As previously noted, this concept refers to the use of science to strengthen and enhance relations among states by fostering extensive networks of collaboration and scientific cooperation. Davis and Patman (2015) cite examples of this modality of science diplomacy, including scientific cooperation agreements, scholarships offered to foreign students, and the creation of international research institutions. This type of arrangement involves a complex network of actors: universities, funding agencies, scientists, and bureaucrats from the countries involved work together across multiple stages of projects, and their roles are not always clearly defined. The synergy generated by scientific cooperation contributes to diplomatic rapprochement among countries. Strouk and Maisonobe (2024) also provide a concrete example of science for diplomacy dynamics, showing how scientific activities in the Svalbard Archipelago serve not only to advance research but also to facilitate the political integration of non-Arctic states into regional governance and cooperation networks.

According to Young et al. (2020), science for diplomacy remains one of the least explored concepts in the science diplomacy literature. While the practice itself is clearly recurrent in interactions between scientists and governments, further studies are needed to consolidate this category, and the present work seeks to contribute to this effort.

### 2.2 Case study: IMT and the ESA's PLATO mission

The Mauá Institute of Technology is a private Brazilian university whose main campus is located in the city of São Caetano do Sul, in the state of São Paulo. Founded in 1961, IMT offers 17 undergraduate programs and is engaged in both teaching and scientific and technological research. The Laboratory of Embedded Electronic Systems (NSEE) is a research laboratory at IMT, established in the late 2000s to work on embedded systems and critical systems, with direct applications in the aerospace sector. Currently, the laboratory comprises approximately 30 members, including seven faculty researchers, three full-time researchers, five graduate fellows, seven interns, as well as volunteer students and undergraduate research fellows. The laboratory is organized into research lines with international collaborations, one of which focuses on aerospace technologies and astronomy, within the context of participation in the PLATO mission. In general, public higher education institutions tend to have greater resources for research, particularly in the aerospace field, which is underfunded in Global South countries. In this regard, IMT represents an exception and collaborates with public universities.

The PLATO mission, an acronym for PLAnetary Transits and Oscillations of stars, is an ESA scientific mission whose primary objective is to launch, by the end of 2026, a satellite equipped with 26 cameras designed to capture images of the space. The mission aims to detect and study multiple extrasolar planetary systems, focusing on investigating the properties of Earth-like exoplanets located in the habitable zones of Sun-like stars (European Space Agency, 2025), which, like Earth, may contain liquid water and, consequently, life forms. The project is structured around an international consortium bringing together scientists from 23 countries, each responsible for a specific scope of work. Funding is provided by ESA member states, which explains the pre-dominance of European countries among the participants (PLATO Mission Consortium, 2025). Nevertheless, the presence and contributions of three Global South countries, Brazil, Chile, and India, stand out. This type of arrangement depends on diplomatic coordination among members, as technical and financial decisions are made collectively.

**Figure 1:**
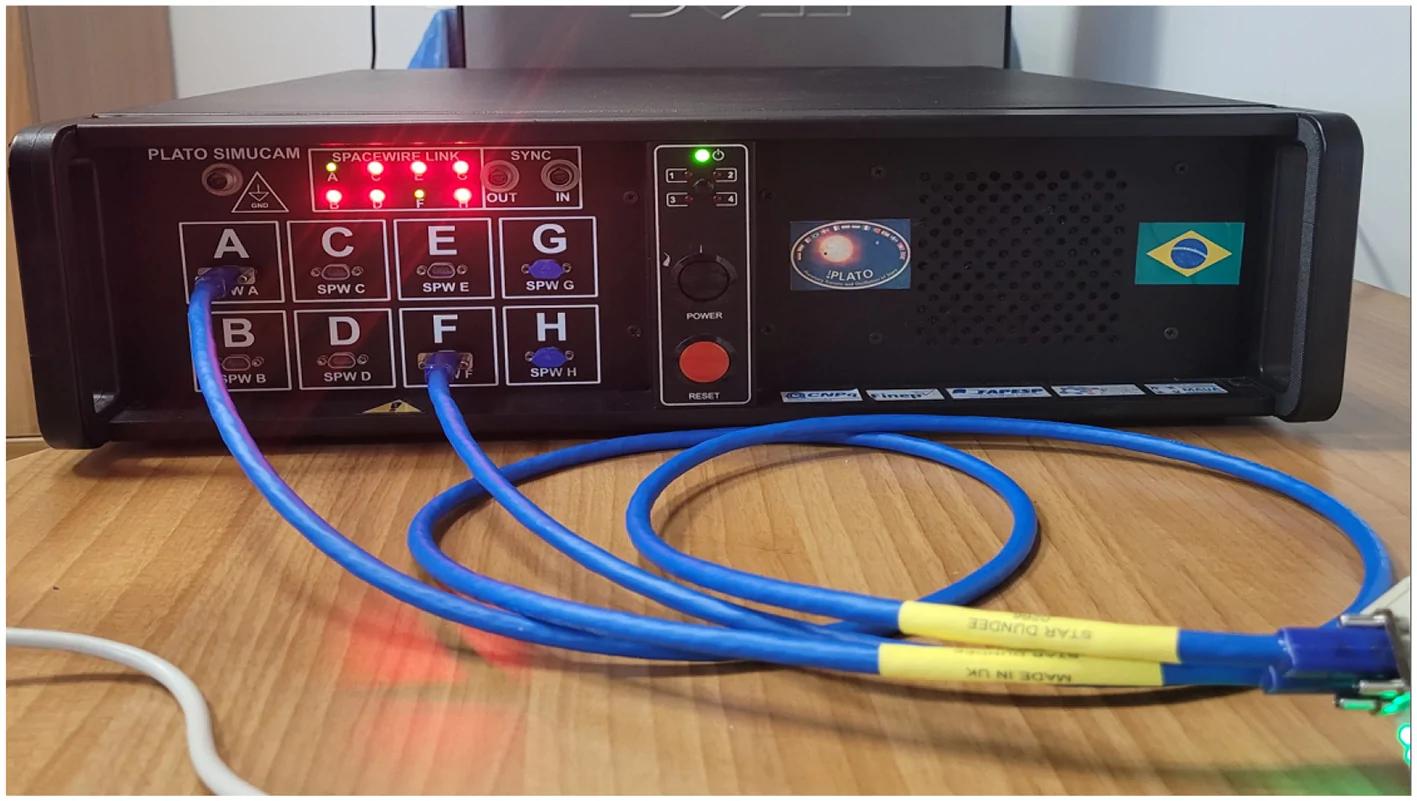
PLATO mission cameras simulator developed by IMT: SimuCam.

IMT's participation in the PLATO mission took place through the development and construction of a device responsible for simulating the operation of the satellite's cameras. This simulator, named SimuCam and shown in Figure 1, operates by transmitting real-time data from the cameras to other onboard computers via a specific space communication protocol used by ESA. In this way, if one of these simulators is replaced by an actual camera, the other interconnected systems will not detect any difference. The presence of a simulator with these characteristics represents a strategic advantage for the mission, since the various onboard computers and software systems were developed in parallel with the cameras. In this context, the SimuCam device plays a crucial role by enabling other teams involved in the PLATO mission to test and validate their systems and software under development, without having to wait for the completion of the cameras.

Among all the countries and teams participating in the PLATO consortium, IMT developed the simulator to meet the needs of five institutes: the OBSPM, France; the Institute of Astrophysics of Andalusia (IAA), Spain; the DLR, Germany; the Space Research Institute (IWF), Austria; and the National Institute for Astrophysics (INAF), Italy. The work was carried out in a parallel and collaborative manner with each of these institutes, requiring consideration of different applications, technical requirements, and also the cultural particularities of each country involved.

By delivering a mission-critical piece of equipment, IMT established itself as a key contributor to the project and was subsequently invited to participate in new scientific initiatives, such as EnVision and VERITAS, both of which are aimed at investigating the planet Venus.

Over more than a decade, the project has been marked by numerous events and significant milestones that reflect both the process of technological development and the continuous strengthening of ties between IMT, national institutions, and the partner institutes and countries previously mentioned. A detailed timeline of the most significant events is presented below.

### 2.3 Timeline

In order to present, in a structured manner, the chronology of events related to IMT's participation in the PLATO mission, the main milestones of each year of cooperation were selected and categorized according to the following four classifications:

- **Six project milestones:** represent technical progress achieved by IMT within the PLATO mission, including the beginning of new project phases, deliveries, scope expansions, or conducted tests.
- **Five institutional milestones:** denote forms of institutional support provided to IMT in the context of the PLATO mission, most often in the form of funding or financial support.
- **Four diplomatic milestones:** refer to moments of diplomatic interaction and rapprochement among the countries involved in IMT's participation in the PLATO mission, typically mediated by governmental institutions.
- **Two milestones representing new opportunities:** refer to invitations to participate in new projects as a result of IMT's work carried out within the PLATO mission.

**Figure 2:**
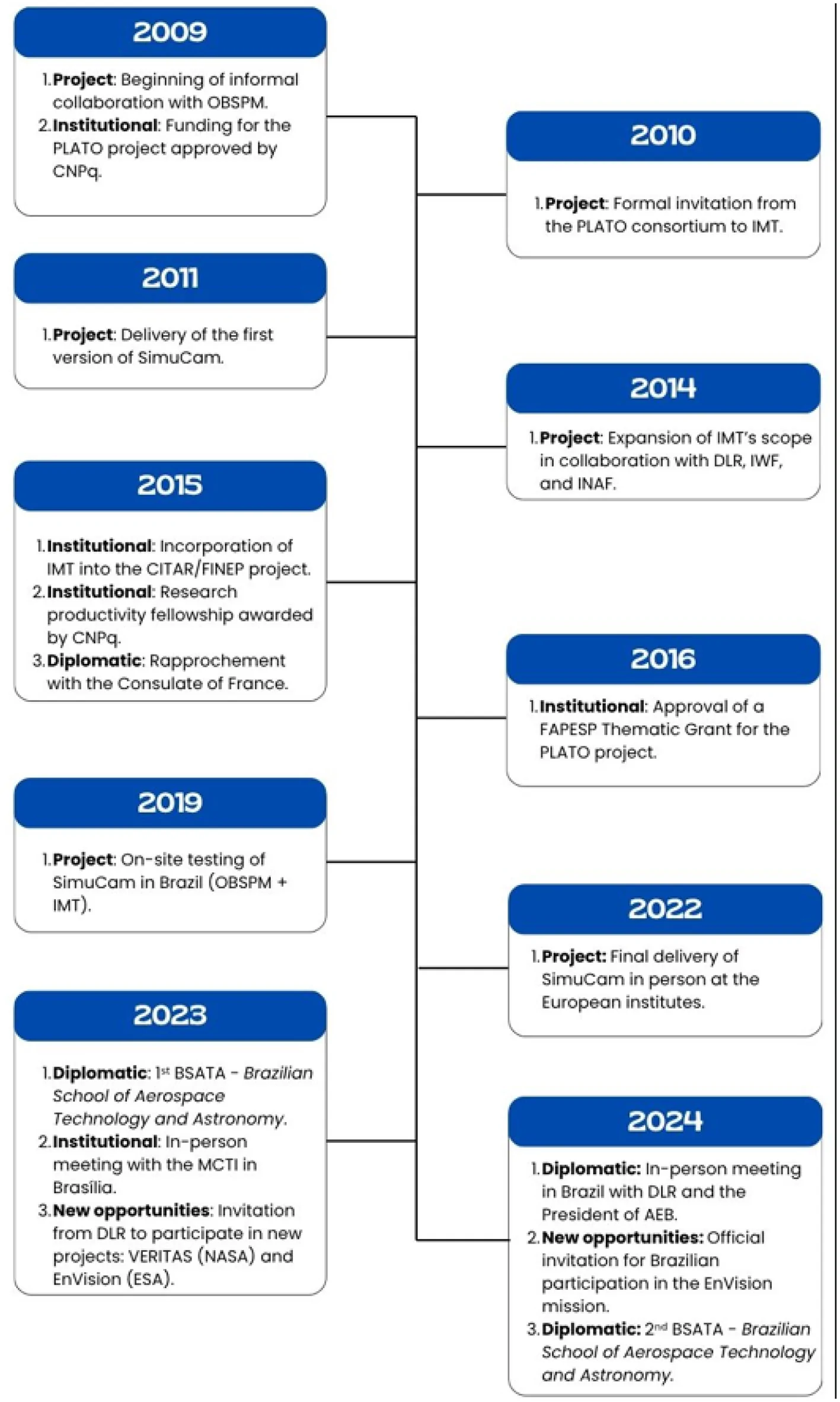
Structured timeline of Brazilian participation via IMT in the ESA PLATO mission, including four types of milestones: project, institutional, diplomatic, and new opportunities.

Comprising these categories of milestones, Figure 2 provides a visual synthesis of the structured timeline of the case study analyzed in this article.

The project began in 2009, when the engineer responsible for the flight software of PLATO's normal cameras^1^, affiliated with OBSPM in France, presented to the lead researcher of NSEE the need to develop a camera simulator and proposed that IMT undertake this task. The prior relationship between the two, established through earlier collaborations, facilitated the initiation of cooperation. Still in 2009, IMT's participation in the PLATO mission resulted in its integration into the National Institute of Space Science and Technology (INEspaço), a Multi-institutional initiative recently approved by Brazil's National Council for Scientific and Technological Development (CNPq), which provided the first funding allocated to the development of SimuCam. The involvement of state funding agencies progressively brought the Brazilian state closer to the project, strengthening the network of scientific cooperation.

In 2010, the CNES, then leading the PLATO consortium, formally invited IMT to develop the satellite's camera simulators. In the same year, French representatives of the mission visited Brazil to hold a seminar on the project. In addition to fulfill the needs of the OBSPM team, the simulators would also be used by its scope partner, IAA in Spain. One year later, in 2011, IMT researchers traveled to Paris to deliver the first version of SimuCam to OBSPM. In that same year, however, the PLATO mission was internally rejected by ESA and subsequently put on hold.

Three years later, in 2014, following technical revisions^2^ proposed by the consortium to ESA, the PLATO mission was resumed under the German leadership of DLR, which formally requested IMT to resume its work and expand its scope to include two new applications: one for DLR, and another for IWF and INAF.

In 2015, IMT was invited to join the CITAR project, led by the Renato Archer Information Technology Center (CTI) and funded by FINEP^3^, both state institutions linked to the MCTI. In the same year, the approval of a research productivity fellowship by CNPq, combined with support from the CITAR/FINEP project, provided institutional and financial backing for the advancement of SimuCam's development within the PLATO project. Also in 2015, the strengthening of IMT's relations with French institutions led to rapprochement with the Consulate of France in São Paulo, which began to follow the initiative and promote outreach materials on scientific cooperation between Brazil and France.

In 2016, IMT partnered with the Institute of Astronomy, Geophysics and Atmospheric Sciences (IAG) at the University of São Paulo (USP), along with other institutions, securing approval from the São Paulo Research Foundation (FAPESP)^4^ for a thematic project that ensured full funding for IMT's scope of work in the PLATO mission.

After 3 years of intensive work, in 2019, the French OBSPM team traveled to Brazil to conduct the first integration tests with SimuCam. These in-person tests demonstrated that European researchers were willing to travel to Brazil to work side by side with Brazilian researchers. Three years later, in 2022, IMT researchers traveled to Europe to complete the final delivery of the equipment to their international partners: OBSPM in France; DLR in Germany; INAF in Italy; and IWF in Austria. This stage marked the conclusion of SimuCam's development phase and the beginning of a new period focused on technical support and maintenance of the equipment until the mission's completion.

In 2023, IMT hosted, on its campus, the first edition of the Brazilian School of Aerospace Technology and Astronomy (BSATA), organized in partnership with CTI Renato Archer, the National Institute for Space Research (INPE), and USP. The event included the participation of researchers and collaborators from OBSPM, DLR, and IWF, who delivered lectures to the Brazilian scientific community and clearly contributed to strengthening ties between foreign and Brazilian actors. In the same year, an in-person meeting with MCTI in Brasília symbolized the consolidation of this rapprochement with the state apparatus within the framework of PLATO mission cooperation. Also in 2023, the success of SimuCam and the trust built over the years led DLR to informally invite IMT to develop emulation and simulation solutions for two new space missions: VERITAS, by NASA, and EnVision, by ESA.

In 2024, DLR officially invited IMT to join the EnVision mission, proposing the adaptation of SimuCam to simulate the spectrographs onboard the satellite. At this new stage, Brazilian participation reached a higher level, as Brazil, through IMT, gained representation by a scientist within the mission scientific decision board. The development of the new simulator will be carried out in direct cooperation with DLR and the Max Planck Institute, a German research institution. As a result of this achievement, both scientific and diplomatic, in-person meetings were held in Brasília in the same year, with the participation of representatives from MCTI, the president of AEB, and a DLR delegate, reinforcing institutional engagement between the countries. These meetings demonstrate that cooperation evolved beyond interactions between laboratories to involve governments. Also in 2024, IMT hosted the second edition of BSATA, an event with both scientific and diplomatic objectives, which once again brought together members of DLR, the Max Planck Institute, and various Brazilian institutions, further strengthening international scientific and technological cooperation ties.

### 2.4 Document-based analysis

The document analysis was conducted through a qualitative and interpretative approach, seeking to identify recurring patterns of international cooperation, institutional trust-building, the expansion of scientific networks, and the gradual rapprochement between academic and governmental actors. In this regard, the documents were not treated merely as administrative or evidentiary records, but rather as evidence of the institutional evolution of the cooperation over time. The recurrence of formal expressions of recognition, invitations to participate in new missions, expansions of technical scope, and the increasing involvement of governmental institutions was interpreted as indicative of the consolidation of relationships among the partners participating in the mission.

The documents, listed in Table 2, also made it possible to observe the gradual transformation of a cooperation initially centered on specific technical demands into a broader network of international collaboration involving universities, research laboratories, space agencies, and governmental institutions. The presence of Brazilian and European state actors at different stages of the cooperation revealed the project's institutional expansion over the years, particularly through the growing interaction among IMT, MCTI, AEB, OBSPM, DLR, and other partner institutes within the PLATO mission.

**Table 2:** Documents received by IMT in the context of the PLATO mission, categorized by the type of actors involved.

Institution	Year	Interaction category	Responsible individual or sender	Main topic addressed
CNES	2012	Foreign state actor in collaboration with IMT	- Data Processing System Manager for PLATO Payload	- Recognition of IMT's contribution to the PLATO mission
AEB	2013	Brazilian state actor in collaboration with a foreign state actor	- President of AEB	- Support for the participation of the Brazilian scientific community in the PLATO mission
OBSPM	2014	Foreign state actor in collaboration with IMT	- Project Manager for the flight software of the Normal cameras Data Processing Units of the PLATO mission	- Recognition of IMT's successful cooperation with OBSPM since 2009
	2018			- Recognition of IMT's successful cooperation with OBSPM, IAA, DLR, and IWF
	2021			- Recognition of IMT's successful cooperation with OBSPM, IAA, DLR, and IWF
				- Certification of the first official delivery of SimuCam
2024			- Recognition of IMT's successful cooperation with OBSPM, IAA, DLR, IWF, and INAF
			- Statement of OBSPM's interest in cooperating on new projects
DLR	2014	Foreign state actor in collaboration with IMT	- Manager of the Data Processing System of the PLATO mission - Leader of the PLATO mission consortium	- Statement of the consortium's interest in cooperation with IMT, including the new scope proposed by DLR
	2021	Foreign state actor in collaboration with IMT	- Manager of the Data Processing System of the PLATO mission	- Recognition of IMT's contribution to the PLATO mission
	2024			- Recognition of IMT's contribution to the PLATO mission
				- Statement of DLR's interest in cooperating on the VERITAS and EnVision missions
2024	Foreign state actor in collaboration with IMT	- VenSpec Suite Coordinator of the EnVision mission	- Official invitation to IMT to participate in the EnVision mission
FAPESP	2016	Brazilian state actor in collaboration with IMT	- Electronic document	- Identification of the FAPESP funding process
CTI Renato Archer	2022	Brazilian state actor in collaboration with IMT	- Coordinator of the CITAR/FINEP project	- Recognition of IMT's cooperation with CTI Renato Archer within the context of the CITAR/FINEP project and the PLATO mission

At the same time, it is acknowledged that the documents analyzed possess limitations inherent to their institutional nature. As they were produced by the actors directly involved in the cooperation (primary sources), many records tend to emphasize the positive outcomes of the project and may reflect specific institutional interests. Accordingly, the documents were examined critically, taking into account the possibility of institutional bias and avoiding their treatment as neutral or definitive evidence of the diplomatic impacts of the cooperation. Thus, document analysis was employed not as conclusive proof of diplomatic outcomes, but as a tool for identifying mechanisms of international interaction, processes of institutional rapprochement, and potential developments arising from the scientific cooperation carried out within the context of the PLATO mission.

## 3 Results

IMT's consolidated participation in the PLATO mission has generated a series of relevant insights into the scientific participation efforts of Global South countries. To facilitate the understanding of the findings of this case study, they were classified into two main dimensions: technical and diplomatic.

### 3.1 Technical results of IMT's participation in the PLATO mission

As previously noted, the development phase of SimuCam has been completed, and IMT has entered the stage of technical support and maintenance, assisting partner institutes whenever necessary. A total of 22 SimuCam units were produced, of which six are located in Brazil and the remainder in the previously mentioned European institutes. These simulators represent Brazilian technology used by renowned European institutions and researchers, demonstrating the potential of the Global South to meet advanced scientific and technological demands. Furthermore, the construction of these devices involved the national industry, which was responsible for manufacturing and supplying specific electronic components for the project, companies that also exported these components so that European laboratories could assemble the simulators. From IMT's side alone, the cooperation resulted in the submission of six intellectual property claims related to software, circuits, and simulator topologies, including the patenting of the SimuCam architecture itself.

Another aspect that should be highlighted as an outcome of the cooperation is the technology transfer carried out by ESA and the European institutes to the Brazilian community involved. The technology transfer occurred through extensive project requirement documentation. IMT's partners defined their needs, and Brazilian engineers and researchers developed solutions that met these specifications. Subsequently, European laboratories, and ESA contractors such as Airbus, conducted extensive testing of the delivered systems to ensure compliance. This process was iterative and continuous throughout the cooperation, resulting in significant human resource development within the Brazilian aerospace community, as it enabled the acquisition of globally validated technical expertise.

A final technical outcome of major relevance concerns access to scientific data derived from participation in the mission. Considering that ESA missions are funded by public resources from member states, there are restrictions on remunerating institutions outside the European bloc, except in exceptional cases. In this context, the cooperation established between IMT and the PLATO mission was strategically structured. IMT committed to developing the satellite's camera simulator in exchange for access to the exclusive scientific data generated by the mission. This arrangement will enable Brazilian astronomers and astrophysicists to conduct cutting-edge research using unprecedented datasets, which remain restricted to the scientific team for a period before becoming publicly available. In this way, Brazil consolidates its integration into the full cycle of scientific space missions, participating not only in technological development but also in the exploration and analysis of results, whose ultimate purpose is the production of knowledge and the advancement of science.

### 3.2 A case of science for diplomacy

Cooperation between IMT and the European institutions involved in the PLATO mission was based on fluid and continuous interaction among all parties. As noted, the development of SimuCam involved a recurring process of meetings to discuss technical matters and requirements, in which each laboratory presented the needs of its specific application. This continuous workflow led to closer ties between Brazilian and European teams, initially at the interpersonal level, and subsequently at the institutional and state levels.

According to the CILAC and UNESCO (2020) report, science diplomacy may be explicit or implicit, and many activities can be considered instances of science diplomacy even without being explicitly labeled as such. In this context, one of the outcomes of the case study presented in this article is the characterization of an episode of science for diplomacy. As cooperation progressed, Brazilian state institutions increasingly became involved in the project, providing various forms of institutional support, whether symbolic or financial. Among these, one may highlight FAPESP, representing the state of São Paulo, as well as MCTI itself and several institutions affiliated with it, such as CNPq, FINEP, CTI Renato Archer, INPE, and AEB. In the final stages of the project, the success of SimuCam and the strong relationship between IMT and foreign state institutions also contributed to bringing these actors closer to Brazilian state institutions.

**Figure 3:**
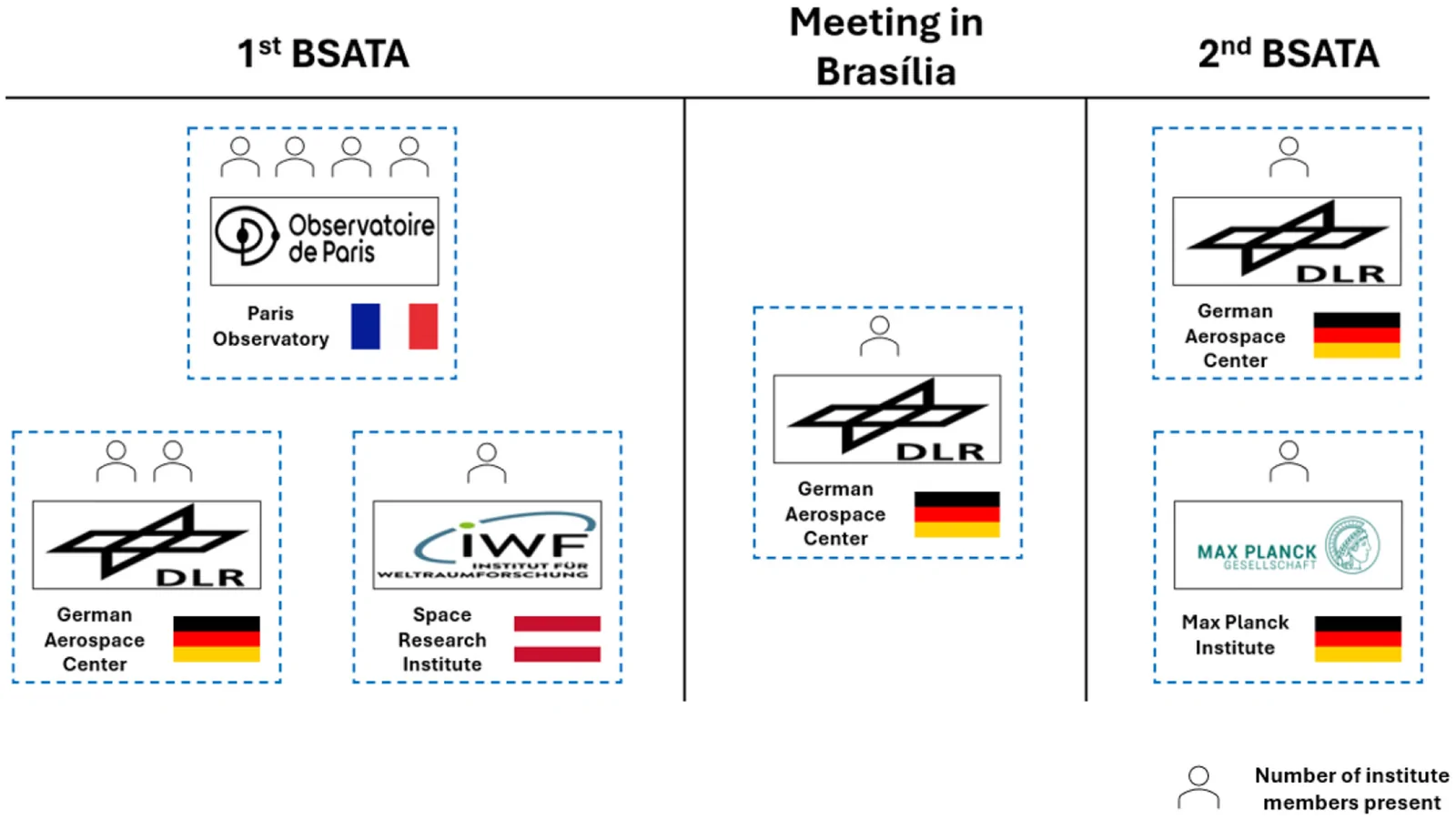
Foreign institutes and the number of representatives present at each of the three science diplomacy milestones.

Although there had been an earlier rapprochement with the Consulate of France in 2015, the 2023 edition of BSATA can be considered the first of three major science diplomacy milestones, as presented in Figure 3. The event, led by IMT, took place on its campus in São Caetano do Sul and was organized jointly with CTI Renato Archer, INPE, and USP, with financial support from FAPESP to cover travel expenses for foreign participants. During the event, representatives from the German and Austrian space agencies, DLR and IWF, as well as research laboratories from France, represented by OBSPM, traveled to Brazil to participate in a series of lectures, visits, and dialogues alongside members of Brazilian institutions. This engagement resulted in a shared intention among all parties to continue cooperating on space projects, including the adaptation of SimuCam for other missions. Additionally, the productive discussions held during the event led, a few months later in 2024, to the second milestone: a joint visit by IMT and a DLR representative to Brasília, where meetings were held with the president of AEB and other members of MCTI to discuss Brazil's participation in the VERITAS and EnVision missions. Shortly thereafter, Brazil was officially invited, through SimuCam adaptation and IMT, to join ESA's EnVision mission. This time, as previously noted, the arrangement included the participation of at least one Brazilian researcher within the scientific team responsible for mission decision-making, the VenSpec Science Team.

At the end of 2024, the second edition of BSATA was held, with presence of representatives from DLR and the Max Planck Institute, new German partners within the framework of the EnVision mission. This final milestone marked the closure of a cycle resulting from years of collaboration between Brazilian and German institutions in the PLATO project, securing a Brazilian institution participation in another high-level space mission, EnVision.

## 4 Discussion

### 4.1 Brazilian institutional constraints and structural North–South aymmetries in international scientific cooperation

A project of the scale of the PLATO mission entails a range of nuances across diplomatic, political, technical, and financial domains. By involving numerous state actors, the composition of the mission consortium becomes vulnerable to political and budgetary fluctuations specific to each member country. Moreover, the construction of a satellite of this magnitude undergoes an ESA-mediated approval process subject to rejection, as evidenced by the project being frozen in 2011 and later resumed in 2014 following a reduction in scope from 32 to 26 cameras. These characteristics mean that a robust presence in the project depends on the integration of state institutions, with political reach, and technical executors.

During the years in which IMT cooperated with ESA and institutes from Germany, France, Austria, Italy, and Spain, the national institutional support received was consistently limited, and representatives linked to the Brazilian government were not directly involved in the project. Despite certain financial contributions, numerous online and in-person meetings were held with minimal participation from government representatives, who could have leveraged these opportunities to engage in broader institutional discussions. In this sense, while the Brazilian federal government financed IMT's participation through CNPq and the CITAR/FINEP project, the potential to strengthen relations with the European countries involved was nationally underutilized. The presence of Brazilian ministerial representatives could have increased the relevance of Brazil's participation and opened additional paths for cooperation. There remains a lack of a cohesive strategy within Brazilian institutional frameworks to leverage scientific cooperation as a tool for diplomatic engagement. It is important to note that, as demonstrated by Awasthi et al. (2023) through cases of plastic pollution policy, including a Brazilian case study, the gap between policy intentions and effective implementation is not unique to S&ID. Rather, the misalignment between formal policy aspirations and subsequent institutional action constitutes a recurring challenge across a wide range of policy domains.

It is argued that greater proactivity on the part of Brazilian governmental institutions would have helped to formally consolidate Brazil's presence within the PLATO consortium, rather than limiting national representation to IMT and its university partner, USP. In this way, a broader scope of engagement could have been pursued, potentially opening new opportunities for participation in other missions through coordinated political and diplomatic action at the state level. The limited institutional participation of Brazilian bureaucratic actors has also been observed across Latin America, as noted by da Silva et al. (2021), who report that the demand from scientists and companies for greater interaction with governmental actors has been increasing, yet remains insufficiently structured.

From a budgetary perspective, the resources allocated for the execution of the project were primarily obtained through research funding from the state-level agency FAPESP, which covered a significant portion of the costs associated with the development of SimuCam, including the acquisition of electronic components and researchers travel missions. Additional funding was provided by CNPq, through research productivity fellowships, and by the CITAR project, financed by FINEP, which supported the acquisition of equipment. Nevertheless, it is important to note that the availability of funding varies depending on the governmental actor involved, and financial limitations may arise, as observed in the interaction with AEB in the context of both the PLATO project and the subsequent cooperation in the EnVision mission. In both cases, AEB stated from the outset that, given its primary mandate as a regulatory and coordinating body, it would not be able to allocate financial resources to support the initiatives, highlighting limitations in the capacity of the agency, which, in principle, should have greater financial reach as the primary national body responsible for aerospace projects.

A central challenge for international cooperation between Brazil and Europe, representing the Global South and the Global North, lies in the compatibility of financing processes. While Europe possesses a highly structured system tailored to aerospace research, governed by national agencies led by ESA, this framework rigidly regulates state financial reserves, local industry reinvestment rules, and project execution by laboratories and companies. Given the system's significant restrictions on financing non-ESA member states, European projects benefit from a predictable lifecycle that extends from the initial call for proposals to a satellite's atmospheric re-entry, a continuity of support and planning that remains inaccessible to actors from the Global South.

In the Brazilian scenario, although the AEB serves as an important regulatory body, funding acquisition is decentralized and relies heavily on general subjects funding agencies. This fragmented institutional configuration fails to guarantee the continuity of virtuous projects. Space research inherently requires accepting technological risks, which can only be mitigated by frameworks that value legacy through long-term stability. Budgetary discontinuity discourages researchers, students, and the private sector, leading to isolated and short-lived initiatives.

Furthermore, there is a pronounced fragmentation of funding and a subsequent increase in bureaucracy per unit invested in core activities. From a comparative perspective, the relative cost of resource acquisition in Brazil is significantly higher than in Europe, as spreading funds across multiple small-scale projects reduces the institutional efficiency that a single large-scale investment would provide. Domestic funding agencies often exhibit a systemic limitation in evaluating aerospace projects, a limitation rooted in specific structural features of the field that distinguish it from more conventional academic research. Aerospace development is inherently legacy-based, building incrementally on decades of accumulated institutional knowledge rather than discrete, self-contained studies. Projects of this scale typically involve hundreds of individuals across dozens of institutional actors, with a substantial share of technical outputs remaining unpublished due to non-disclosure agreements protecting proprietary or strategic information. Furthermore, the intensive technology development inherent to the field is frequently carried out by engineers and undergraduate or technical students rather than being channeled through formal master's or doctoral thesis production, the primary output traditionally rewarded by academic evaluation frameworks. As a result, conventional peer-review criteria, largely designed around individually attributable, published academic research, are structurally ill-suited to capture the value of this type of work, leading to inconsistent assessments that, rather than guiding research improvement, induce conceptual confusion among evaluators and applicants alike. Such friction undermines a healthy institutional ecosystem where funding bodies should act as catalysts for aerospace advancement. Consequently, this leads to a fragmentation of research agendas, decentralized procurement, and a proliferation of redundant, small-scale initiatives trying to navigate the arduous pathway required by space missions.

Structural asymmetries also exist in the stock and distribution of knowledge among universities, space agencies, and industry. In the European model, the roles of each actor are clearly delimited, minimizing institutional overlap and maximizing the circulation and availability of technical knowledge, as exemplified by CNES's TTVS course, which serves as a hub for knowledge dissemination. In contrast, throughout much of the Global South, the absence of a robust domestic aerospace and defense industry frequently compels universities to assume responsibilities typically assigned to industrial actors. In Brazil, this challenge is further exacerbated by overlapping jurisdictions and unclear divisions of responsibility among institutions such as the AEB, INPE, and the Brazilian Air Force. Paradoxically, however, these structural constraints have also fostered distinctive capabilities. Whereas the European model tends to maintain a strong separation between scientific research and engineering activities, ensuring a high and positive degree of specialization but potentially creating barriers to system integration, Brazilian teams, partly in response to institutional and resource constraints, have developed a greater capacity to operate across institutional and technical boundaries. As a result, within large-scale international consortia, Brazilian participation is frequently associated with flexibility, adaptability, and problem-solving capabilities that help bridge integration gaps between specialized domains. Although this socio-technical dynamic warrants further empirical investigation, the 20-year history of sustained collaboration presented in this study provides a strong preliminary indication of an asymmetric complementarity in which the institutional constraints of the Global South have generated capabilities that effectively complement the highly and well specialized structures characteristic of the Global North.

A critical and under-theorized dimension of the type of cooperation discussed in this work is its transactional model: a barter system where Global South institutions deliver concrete technology in exchange for exclusive scientific data access. Participating in a €1.5 billion mission such as PLATO grants Brazilian astronomy direct access to cutting-edge datasets. However, measuring the economic and tangible returns of this data-for-technology exchange remains an open debate within Brazilian science policy circles, as well as among stakeholders within IMT itself. This controversy exposes a broader systemic friction in domestic funding mechanisms and institutional support structures, again including those present within IMT.

Frequently, the professional profile of an aerospace engineer capable of delivering a sensor-simulation instrument of this magnitude does not align with the strictly academic evaluation criteria used by traditional funding agencies. To bypass this barrier and secure funding, technological developers must early on embed their projects within purely scientific agendas. This workaround highlights a profound structural limitation in evaluating technological research. Furthermore, it reveals an institutional failure to recognize that contemporary high-impact science, “Big Science,” is intrinsically driven by large, multi-author consortia. It is not uncommon for project proposals in Brazil to face negative peer-review assessments due to co-authored publications with extensive lists of contributors, where reviewers diminish the research's value because they struggle to isolate the individual proponent's direct share. Paradoxically, this individualistic metrics framework ignores the immense geopolitical and scientific capital of international integration. Being one among dozens of co-authors in a global space consortium represents a highly exclusive and strategic institutional achievement, excluding tens of thousands of other global actors.

Moreover, while innovation and intellectual property protection are nominally recognized as merit indicators, they often prove insufficient to secure research project funding within traditional academic frameworks. Scientific papers remain the predominant currency of peer-review panels. This bias systematically ignores the fact that a technical report delivered to and approved by an agency like ESA undergoes dozens of expert reviews, a process that is as rigorous as standard journal peer review.

Another overlooked aspect is the intrinsic technology transfer capacity embedded within these cooperative frameworks. Space missions inherently demand high levels of maturity for the developed hardware and software elements, directly pushing them up the Technology Readiness Level (TRL) scale. The underlying mechanism is systemic: a partner country requiring a specific technological solution must explicitly formalize detailed specifications, which naturally triggers an initial technology transfer. The executing party absorbs the operational costs of this process but, in return, trains personnel, refines institutional processes, and upgrades corporate capabilities. Once the solution is delivered, transferring the technology forward, the receiving international agency validates the output. This institutional validation generates a high reputation “seal of approval,” which effectively transfers technical validation back to the developer. Utilizing these assets in continuous testing and verification environments fundamentally enhances the solutions, systematically accelerating their TRL advancement.

Notwithstanding, this cooperation process should also be interpreted through the lens of power asymmetries between the Global South and the Global North. Given that sophisticated systems, technologies, and equipment are being developed within these collaborations, it is not unreasonable to argue that financial compensation could constitute an equitable form of contribution. However, the limited political, economic, and geopolitical leverage of many Global South countries often places them in a structurally subordinate negotiating position, although this statistical condition did not characterize the project's collaborative dynamics and involvement evolution. Moreover, many European institutions, unlike many Brazilian ones, operate within legal and administrative frameworks that do not provide mechanisms for financial compensation, including financial support for project expenses of external partners outside the European bloc, thereby constraining the range of possible arrangements available within international collaborations. As a result, access to scientific datasets and opportunities for technology transfer frequently become the primary currencies of exchange deemed sufficient to sustain cooperation. While such arrangements generate important scientific and technological benefits, they also reflect broader asymmetries in the international distribution of resources, bargaining power, and decision-making authority that continue to shape North–South scientific partnerships.

### 4.2 The role of international cooperation among researchers in the field of science diplomacy

Although the case study achieved several objectives related to technological development, particularly in the fields of engineering and astronomy, one aspect that stood out as especially positive was the act of science diplomacy carried out through international cooperation among researchers, highlighting the novelty of the topic.

Science diplomacy, due to its direct connection to foreign policy, is commonly viewed as a responsibility of the state, with governmental apparatuses acting as its primary executors. However, research in this recently established field demonstrates that numerous actors, both state and non-state, are capable of practicing science diplomacy. Two excerpts from the (CILAC and UNESCO, 2020, authors' translation), strongly support this perspective, noting that “scientists working in universities and other non-governmental institutions may often not recognize their actions in relation to science diplomacy, yet they may act as active agents of it.” This point is significant, as it highlights both the absence of a structured science diplomacy strategy within state bureaucracies and the limited awareness of the topic among technical communities. Training initiatives are therefore necessary to integrate science diplomacy into the everyday practices of scientists and bureaucrats, enabling opportunities for scientific cooperation to also be leveraged in the diplomatic sphere, thereby generating synergy among the different actors involved. As emphasized in the (CILAC and UNESCO, 2020, authors' translation) report: “(...) science diplomacy is driven not only by states, but there is an increasing number of cases involving cities, regional governments, universities, multinational companies, and other non-state actors.”

One of the key findings of this specific single-case study reveals that, when examining the trajectory of the cooperation, a spontaneous case of science for diplomacy can be identified. IMT's initial interaction with European institutes occurred exclusively through the personal and academic contact of a faculty researcher from the university. From that point onward, subsequent relationships within the context of the PLATO project continued to develop on a personal basis, and positive recommendations regarding IMT and its members attracted the interest of other international institutes in the work carried out by the Brazilian team. In this way, a process of rapprochement was established that evolved naturally, in parallel with the project's technical progress.

Throughout the cooperation, there were significant milestones in which Brazilian governmental institutions met with their foreign counterparts, most notably the DLR meeting in Brasília with the president of AEB, as well as the first and second editions of BSATA. In these encounters, which became more frequent especially in the final phase of the project, a degree of inter-state rapprochement emerged through the exchanges and discussions held during meetings and events, elevating the level of cooperation as some formal representation of the state actors involved became consolidated. Not coincidentally, it was following two key diplomatic milestones, the first BSATA and the meeting in Brasília with AEB, that the official invitation emerged for IMT, and consequently Brazil, to participate in scientific decision-making level, the VenSpec Science Team, in a new mission, EnVision.

The analysis of the case made it possible to identify a set of mechanisms that worked on this single-case, through which a scientific cooperation initiative that was initially technical in nature came to generate broader institutional effects, and a few diplomatic outcomes. Rather than assuming that scientific cooperation automatically produces diplomatic outcomes, the study suggests that specific intermediary processes facilitated the gradual rapprochement between Brazilian and European actors throughout the PLATO mission.

The first mechanism identified was the recurring technical interaction among researchers and engineers involved in the development of SimuCam. The continuous need for meetings, validations, testing procedures, and joint problem-solving contributed to the construction of trust-based relationships among the participants in the cooperation. This trust, accumulated over the years, facilitated the expansion of interactions beyond the project's initial technical demands.

The second observable mechanism relates to the institutional expansion of the cooperation. As the project progressed and IMT demonstrated its technical capacity to meet the requirements of the PLATO mission, additional European institutes joined the collaboration network. Simultaneously, Brazilian funding agencies and governmental institutions became increasingly involved in the process, whether through financial support, institutional backing, or participation in scientific events associated with the project.

The third mechanism identified involves the creation of hybrid spaces of interaction between scientific and governmental actors. Events such as BSATA and in-person meetings with representatives of AEB and MCTI elevated the institutional level of the cooperation, allowing relationships initially established among researchers to expand and include Brazilian and European state representatives. These encounters contributed to transforming a pre-dominantly technical collaboration into a broader channel for institutional rapprochement.

Finally, the consolidation of these networks of trust and cooperation contributed to the emergence of new international invitations and opportunities for Brazilian participation in other space missions, such as EnVision and Veritas. The study suggests that the diplomatic effects observed did not result from a previously planned formal diplomatic strategy. Rather, they emerged gradually through the intensification of the scientific, institutional, and interpersonal relationships developed throughout the course of the international cooperation.

Nonetheless, looking to the future of the research topic, a recent study by Awan et al. (2025), which analyzes the composition of participants in InnSciD, an S&ID event organized by USP, indicates that more than half of the participants came from academia, followed by government representatives. This finding highlights the growing diffusion of the topic among academics and the increasingly clear recognition of its relevance in both scientific and diplomatic contexts.

## 5 Conclusions

The analysis of IMT's participation in ESA's PLATO mission highlights how academic institutions from the Global South can play an active role in highly complex processes of international scientific cooperation, even in the face of structural and institutional constraints. Over more than a decade of international collaboration, IMT became part of an extensive transnational network involving researchers, laboratories, space agencies, and European and Brazilian governmental institutions, contributing to the development of scientific and technological capabilities in a context historically characterized by asymmetries between the Global North and the Global South.

The findings of this single-case study are examples that suggest that international scientific cooperation can generate institutional and diplomatic effects that extend beyond the initial technical objectives of research projects. In the case analyzed, the recurring interactions among researchers and engineers, the continuous need for technical coordination, and the gradual construction of trust among partners contributed to the expansion of cooperation networks and to the rapprochement between Brazilian and European scientific and governmental institutions. In this regard, the study indicates that the diplomatic effects observed did not result from a previously planned formal foreign policy strategy, but rather emerged progressively through the institutionalization of relationships developed within the framework of scientific cooperation.

The case also highlights the relevance of non-state actors in the contemporary practice of science diplomacy. IMT's trajectory shows that universities, researchers, and laboratories can serve as important international intermediaries, creating lasting connections between countries and contributing to the circulation of knowledge, the development of technical capabilities, and the expansion of the international engagement of Global South institutions. The evolution of the cooperation examined in this study illustrates how relationships that initially emerged informally and were centered on specific technical demands can gradually acquire greater institutional density, involving funding agencies, ministries, space agencies, and new international projects.

Furthermore, the research suggests that participation in large-scale international scientific projects can function as a mechanism of peripheral integration into global networks of knowledge and innovation. IMT's experience in the PLATO mission demonstrates that institutions from the Global South can not only absorb technical knowledge but also actively contribute to frontier scientific projects, thereby expanding opportunities for future cooperation and access to new international research arenas.

At the same time, the study reveals important limitations regarding the institutionalization of science diplomacy in Brazil. Although the project benefited from intermittent support from funding agencies and public institutions, interaction between the scientific community and governmental actors remained relatively limited throughout much of the cooperation process. This finding points to the need for stronger coordination among universities, scientific agencies, and state institutions responsible for research internationalization and the formulation of international scientific cooperation strategies.

The research also presents methodological limitations that should be taken into consideration. As a single-case study, its findings cannot be generalized to all contexts of science diplomacy or international cooperation involving Global South countries. Furthermore, a substantial portion of the evidence analyzed derives from institutional documents produced by the actors directly involved in the cooperation, requiring caution in the interpretation of both the findings and the diplomatic impacts observed.

Finally, this study contributes to an emerging research agenda on knowledge circulation; technology transfer; science diplomacy; knowledge and power asymmetries in Global North-South collaboration; and the internationalization and visibility of science from the Global South. Agenda proposed by the Research Topic “Linking Knowledge Across Borders: Science Diasporas and Global South Development” scope. Future research may deepen comparative analyses of different experiences of peripheral participation in international scientific projects, investigating how political, economic, and technological asymmetries shape the integration of Global South actors into research networks. Further studies are also needed on the mechanisms of trust-building among international scientific partners, the limits of cooperation in contexts of technological dependence, and the role of universities, laboratories, and researchers as diplomatic intermediaries within multilateral environments of science and innovation. In addition, studies employing semi-structured interviews and multiple institutional perspectives may contribute to a more comprehensive understanding of how long-term scientific interactions generate institutional, political, and diplomatic effects across different scales.

## Data Availability

The original contributions presented in the study are included in the article/supplementary material, further inquiries can be directed to the corresponding author.

## Footnotes

1 The PLATO satellite has 24 normal cameras, responsible for scientific images of the space, and two fast cameras responsible for satellite attitude control.

2 PLATO was initially designed as a M2 project (Medium size 2), but was rejected for this satellite category and was approved as a M3 project, with an estimated cost of 1.5 billion Euros.

3 FINEP is a public company created to promote Brazil's economic and social development through public funding for Science, Technology and Innovation at companies, universities, technological institutes and other public or private institutions.

4 FAPESP is a public foundation, funded by the taxpayer in the State of São Paulo, with the mission to support research projects in higher education and research institutions, in all fields of knowledge.
